# The association between effort-reward imbalance and job burnout among emergency nurses: the moderating effect of over-commitment

**DOI:** 10.3389/fpubh.2025.1707511

**Published:** 2026-01-16

**Authors:** Man Li, Jing Zhou, Hao Zhang, Dongmei Diao, Jiao Lei, Li Ma

**Affiliations:** 1 Department of Emergency Medicine, West China Hospital, Chengdu, China; 2 Sichuan University/West China School of Nursing, Sichuan University, Chengdu, China; 3 Disaster Medical Center, Sichuan University, Chengdu, China; 4 Nursing Key Laboratory of Sichuan Province, Chengdu, China; 5 Outpatient Department, West China Hospital of Sichuan University, Chengdu, China

**Keywords:** effort-reward imbalance, emergency nurse, job burnout, moderating effect, over-commitment

## Abstract

**Background:**

Studies have shown a strong correlation between occupational stress and burnout. It is still unclear whether over-commitment moderates the association of effort-reward imbalance on job burnout and the extent of its influence.

**Aim:**

To explore the moderating effect of over-commitment on the relationship between external factors of occupational stress and job burnout among emergency nurses, providing theoretical evidence for clarifying the health effects of over-commitment.

**Study design:**

A cross-sectional survey was conducted using a stratified cluster sampling method to select 1,540 emergency nurses from 30 hospitals in China as the study population. The Effort-Reward Imbalance (ERI) Scale and the Job Burnout Scale were used to assess the current status of effort-reward imbalance and job burnout among emergency nurses. SPSS 26.0 was used for correlation analysis, and the Bootstrap method was employed to verify the moderating effect of over-commitment.

**Results:**

A total of 1,551 questionnaires were distributed, and 1,540 valid questionnaires were recovered. The effort-reward imbalance (ERI) of emergency nurses was 0.93 ± 0.57, and the job burnout score was 4.77 ± 6.16. Correlation analysis showed that effort, over-commitment, and job burnout were positively correlated (correlation coefficients: 0.65 and 0.62, respectively; *p* < 0.01), reward and job burnout were negatively correlated (correlation coefficients: -0.53, *p* < 0.01). ERI was positively correlated with job burnout (*r* = 0.62, *p* < 0.01), and the differences were statistically significant. Moderating effect analysis showed that after controlling for work characteristics such as weekly working hours, frequency of night shifts, and monthly income, adding over-commitment into the model revealed that the interaction term between ERI and over-commitment had a significant negative predictive effect on burnout (*β* = −0.31, *t* = −8.48, *p* < 0.001). At different levels of over-commitment, as the level of over-commitment of emergency nurses decreased, ERI was more likely to lead to job burnout.

**Conclusion:**

Higher levels of over-commitment appear to buffer the adverse effect of ERI on burnout, suggesting a protective moderating role.

## 1 Introduction

Effort-reward imbalance (ERI) refers to the physiological and psychological adverse reactions that arise when an individual’s work demands and personal capabilities or resources are imbalanced under work or work-related factors. Proposed by Professor Siegrist in 1996, this concept encompasses three psychological measurement indicators: effort, reward, and over-commitment. Work demands are represented by effort, while reward includes multiple dimensions such as financial compensation, promotion, respect, status, and job security. Over-commitment refers to excessive effort an individual puts into their work. This model has been widely applied to assess occupational stress across different populations (1–3). Among various occupational stress assessment models, the ERI model posits that occupational stress results not only from work demands (such as workload and responsibilities) but also from an imbalance between effort and reward. Unlike other theoretical models, the ERI model introduces the concept of over-commitment. Compared to external stress factors such as effort and reward, over-commitment is defined as an individual’s endogenous stress factor, which in some aspects reflects the risk of occupational stress caused by personality traits related to work. Research shows that the incidence of effort-reward imbalance among emergency nurses is 72.8%, whereas previous studies reported lower detection rates of effort-reward imbalance among clinical nurses, ranging from 26.5 to 40.6%. Overextended hours, excessive workloads, high-intensity tasks, and stressful work environments trigger occupational stress, leading to decreased work efficiency, higher turnover rates, and reduced job satisfaction.

Job burnout is a psychological syndrome characterized by emotional exhaustion, depersonalization, and reduced professional efficacy. It represents a chronic response to work-related stress, damaging individuals’ cognition, emotions, and attitudes, and severely impacting physical and mental health (4–6). Due to the unique nature of their work, nurses are a high-risk group for job burnout. Statistics show that the incidence of job burnout among psychiatric nurses is 49.12%, while that among pediatric nurses is 65.7%. However, the job burnout incidence among emergency nurses reaches as high as 80–90% (7–9), warranting significant attention. Job burnout can cause headaches, insomnia, memory loss, neurasthenia, gastrointestinal disorders, metabolic syndrome, inflammation, immune suppression, and unhealthy behaviors. It can also lead to adverse emotions such as anxiety, depression, tension, irritability, and aggression. In the workplace, it manifests as decreased concentration, reduced initiative, and lower work efficiency, potentially resulting in medical errors and eventual resignation (9–12). Factors such as excessive workloads, high-pressure environments, low nurse-to-patient ratios, poor collaboration between doctors and nurses, lack of organizational leadership and support, and individual psychological conditions contribute to job burnout among nurses (13–15).

Studies have shown a strong correlation between occupational stress and burnout (16). The study by Yuan et al. found that effort-reward imbalance and over-commitment were significantly positively correlated with emotional exhaustion and cynicism. Effort-reward imbalance was a predictive factor for emotional exhaustion and cynicism, while over-commitment moderated the impact of effort-reward imbalance on emotional exhaustion and cynicism (17). However, current research on the correlation between effort-reward imbalance and job burnout among emergency nurses primarily focuses on external factors (effort and reward dimensions) to examine the impact of occupational stress on burnout. Research on the influence of intrinsic factors (over-commitment dimension) remains limited. It is still unclear whether over-commitment moderates the effect of effort-reward imbalance on job burnout and the extent of its influence.

This study, based on the effort-reward imbalance model, proposes the following three hypotheses: (1) Effort, reward, and over-commitment dimensions independently affect job burnout; (2) Effort-reward imbalance has a stronger impact on job burnout compared to individual effort or reward factors; and (3) Over-commitment moderates the relationship between ERI and job burnout. The study aims to explore the effect of effort-reward imbalance on job burnout among emergency nurses.

## 2 Methodology

### 2.1 Research design

A Cross-sectional Survey Study.

### 2.2 Study subjects

This study adopted a cluster sampling method based on the national geographic regions (Northeast, North China, East China, Central China, South China, Southwest, Northwest). Emergency nurses with a work experience of ≥1 year were included in the study, while emergency nurses who were on sick leave, maternity leave, or breastfeeding leave for more than 1 month were excluded. Based on a significance level of 0.05 and a power of 80%, *n* = *Z*^2^ * *p*(1−*p*)/*d*^2^, the required sample size for this study was calculated to be 1,330 cases, according to the results of a preliminary survey.

### 2.3 Research tools

A self-designed general demographic questionnaire was used, which included information on gender, age, education level, marital status, childbearing status, smoking, alcohol consumption, sleep conditions, years of work experience, weekly working hours, frequency of night shifts, monthly income, and other factors.

The Chinese version of the Maslach Burnout Inventory-General Survey (MBI-GS), translated and adapted by Zhu Wei and others, was used to assess the burnout status of emergency nurses (18, 19). This scale includes three dimensions: emotional exhaustion, depersonalization, and professional efficacy, with a total of 16 items. All items are scored using a 7-point Likert scale, ranging from 0 (Never) to 6 (Every day). The emotional exhaustion and depersonalization dimensions use direct scoring, where a higher score indicates a higher level of burnout, while the Professional efficacy dimension uses reverse scoring, where a lower score indicates a higher level of burnout. The overall burnout score is calculated as: Comprehensive score of job burnout = [0.4 × emotional exhaustion score + 0.3 × depersonalization score + 0.3 × (6 − professional efficacy score)] (20). A total score <1.5 indicates no burnout, a score between 1.5 and 3.5 indicates suspected burnout, and a score ≥3.5 indicates burnout. In this study, the Cronbach’s *α* coefficients for the three dimensions and the total scale were 0.914, 0.814, 0.899, and 0.860, respectively.

The Effort-Reward Imbalance (ERI) model, developed by German scholar Siegrist and adapted into Chinese by Dai Junming, was used to assess the occupational stress of emergency nurses (21, 22). The ERI scale consists of three dimensions and 22 items, with 6 items for the effort dimension, 11 items for the reward dimension, and 5 items for the over-commitment dimension. All items are scored using a 5-point Likert scale, ranging from 1 (Strongly Disagree) to 5 (Strongly Agree). A higher total score indicates a higher level of occupational stress. In this study, the total scale Cronbach’s *α* coefficient was 0.82, and the Cronbach’s α coefficients for the three dimensions were 0.75 (effort), 0.89 (reward), and 0.84 (over-commitment), indicating good reliability. The Effort-Reward Ratio (ERR) was calculated to determine whether there was an imbalance. The Effort-Reward Ratio (ERR) is calculated as (Effort/Reward) × c, where c represents a correction factor. The ERR is treated as a continuous independent variable (ranging from 0.20 to 5.00 in this study). ERR = (Effort score/Reward score) × (11/6). A value close to zero indicates favorable conditions (relatively low effort and relatively high reward), while a value greater than 1.0 indicates high effort with insufficient or unmet rewards. When ERR >1.0, the larger the ERR value, the more severe the high effort-low reward state among emergency nurses.

### 2.4 Data collection method

An electronic questionnaire was developed, which included an informed consent form. All items in the questionnaire were set as mandatory to ensure completeness, and each IP address was limited to one submission to avoid duplicate responses, completing the questionnaire within 3 min. Prior to the survey, approval and support were obtained from the head nurse of the emergency department of each participating hospital. A core coordinator was designated in each hospital and received training on the key concepts and guidance for the survey. During the survey, a QR code link was distributed to the subjects via WeChat, and the purpose of the study was explained to the participants. After reading the informed consent form and agreeing to participate in the study, the participants entered the formal questionnaire interface. A total of 1,551 questionnaires were collected, and after excluding 11 questionnaires that showed regular response patterns for any scale and completed in an unreasonably short duration, 1,540 questionnaires were included in the final analysis.

### 2.5 Statistical methods

Data analysis was performed using SPSS 26.0 software. Categorical data were presented as frequency and percentage, while continuous data were expressed as mean ± standard deviation. Pearson correlation analysis was used to explore the bivariate correlation and direction of associations. The moderating effect is tested using Model 1 of the PROCESS macro in SPSS.

## 3 Results

### 3.1 Characteristics of participants

A total of 1,540 emergency nurses from 30 hospitals were surveyed, with 78.6% of the participants being female. Descriptive analysis of the demographic information, lifestyle habits, and occupational characteristics of the 1,540 emergency nurses is shown in Table 1.

**Table 1:** Baseline characteristic information of the study population (*n* = 1,540).

Variable	Category	*n*	Percentage (%)
Age	20–29	582	37.8
	30–39	734	47.7
	40–49	183	11.9
	≥50	41	2.7
Sex	Female	1,211	78.6
	Male	329	21.4
Marital status	Single	560	36.4
	Married	980	63.6
Fertility status	No	710	46.1
	Yes	830	53.9
Education	Junior college degree	186	12.1
	Bachelor’s degree or above	1,354	87.9
Professional title	Junior	907	58.9
	Intermediate	574	37.3
	Senior	59	3.8
Years of work (year)	≤5	491	31.9
	6–10	493	32.0
	11–15	300	19.5
	16–20	121	7.8
	>20	135	8.8
Weekly working hours (h/week)	<40	577	37.5
	41–48	780	50.6
	49–58	123	8.0
	≥59	60	3.9
Night shift situation	No	181	11.8
	Yes	1,359	88.2
Number of night shifts per month	0 times/week	181	11.8
	1–4 times/week	239	15.5
	5–8 times/week	659	42.8
	≥9 times/week	461	29.9
Monthly income	<4,000 yuan/month	57	3.7
	4,000–5,999 yuan/month	149	9.7
	6,000–7,999 yuan/month	250	16.2
	8,000–9,999 yuan/month	368	23.9
	≥10,000 yuan/month	716	46.5
Smoking situation	Do not smoke	1,453	94.4
	Smoking	87	5.6
Alcohol consumption situation	Do not drink alcohol	1,355	88.0
	Drinking	185	12.0

### 3.2 ERI scores and burnout scores of emergency nurses

The scores for the three dimensions—effort, reward, and over-commitment—were (19.22 ± 5.35), (23.72 ± 9.10), and (12.6 ± 4.74), respectively. The Effort-Reward Imbalance (ERI) was 0.93 ± 0.57, indicating a moderate level of effort-reward imbalance among emergency nurses. The overall burnout score was (4.77 ± 6.16). The scores for the emotional exhaustion, depersonalization, and professional efficacy dimensions were (11.30 ± 7.76), (8.61 ± 7.58), and (13.79 ± 10.04), respectively. Among the emergency nurses, 882 had an overall burnout score ≥3.5, indicating that 57.3% of emergency nurses experienced burnout. A further 327 nurses (21.2%) were classified as having suspected burnout. The results suggest a significant burnout problem among emergency nurses. Specific data on the ERI scores and burnout scores of emergency nurses can be found in Table 2.

**Table 2:** Scale and dimension scores.

Project	Minimum value	Maximum value	Score $\left(\bar{x}\pm s\right)$
Effort-reward imbalance	0.20	5	0.93 ± 0.57
Effort	6	30	19.22 ± 5.35
Reward	11	55	23.72 ± 9.10
Over-commitment	5	25	12.60 ± 4.74
Job burnout	−9	22.8	4.77 ± 6.16
Emotional exhaustion	0	30	11.30 ± 7.76
Depersonalization	0	30	8.61 ± 7.58
Professional efficacy	0	36	13.79 ± 10.04

### 3.3 A multiple regression analysis that includes all three dimensions

Firstly, we conducted univariate analyses to examine the associations between the three dimensions of the ERI and burnout. The results indicated that effort, over-commitment, and reward were all significantly associated with burnout (*F* = 49.94, 55.43, and 20.82; *p* < 0.001, respectively). Variables with statistical significance were subsequently included in a multiple regression analysis (Effort [*B* = 0.49, 95%CI (0.44, 0.55)], Over-commitment [*B* = 0.29, 95%CI (0.20, 0.37)], and Reward [*B* = −0.10, 95%CI (−0.14, −0.07)]), the results of which are presented in Table 3.

**Table 3:** A multiple regression analysis that includes all three dimensions.

Variable	*B*	SE	*P*	95%CI		Tolerance	VIF
				LLCI	ULCI		
Effort	0.49	0.03	<0.001	0.44	0.55	0.56	1.78
Over-commitment	0.29	0.04	<0.001	0.20	0.37	0.32	3.14
Reward	−0.10	0.02	<0.001	−0.14	−0.07	0.42	2.36
*R*	0.70^a^						
*R* ^2^	0.50						
Adjusted *R*^2^	0.50						
Change in *R*^2^	0.50						
*F*	502.25						
*P*	0.001						

Dependent variable: burnout.

a Predictor variable: effort, over-commitment, and reward.

### 3.4 A hierarchical regression analysis incorporating ERI and its three dimensions

This hypothesis was statistically examined using hierarchical regression analysis to assess changes in *R*^2^ as ERI, effort, and reward were sequentially entered into the model. The results demonstrated that ERI exerted a significantly stronger influence on burnout compared to effort or reward alone (*R*^2^ = 0.42, 0.48, 0.49), the results of which are presented in Table 4.

**Table 4:** A hierarchical regression analysis incorporating ERI and its three dimensions.

Variable	*B*	SE	*P*	95%CI		Tolerance	VIF
				LLCI	ULCI		
Effort	0.75	0.02	<0.001	0.70	0.79	1	1
Effort	0.59	0.02	<0.001	0.54	0.64	0.75	1.34
Reward	−0.19	0.01	<0.001	−0.22	−0.16	0.75	1.34
Effort	0.50	0.03	<0.001	0.45	0.56	0.50	1.99
Reward	−0.11	0.02	<0.001	−0.16	−0.07	0.36	2.81
ERI	1.97	0.40	<0.001	1.19	2.75	0.24	4.12
*R*	0.65^a^	0.69^b^	0.70^c^				
*R* ^2^	0.42	0.48	0.49				
Adjusted *R*^2^	0.42	0.48	0.49				
Change in *R*^2^	0.42	0.06	0.01				
*F*	1,121.52	171.97	24.43				
*P*	0.001	0.001	0.001				

Dependent variable: burnout.

a Predictor variable: effort.

b Predictor variable: effort, reward.

c Predictor variable: effort, reward, ERI.

### 3.5 Correlation analysis between effort-reward imbalance and burnout

Table 5 shows that effort and over-commitment are positively correlated with emotional exhaustion (the correlation coefficients for effort and over-commitment with emotional exhaustion are 0.62 and 0.68, respectively, *p* < 0.01), while reward is negatively correlated with emotional exhaustion (the correlation coefficients for reward with emotional exhaustion is −0.63, *p* < 0.01). Effort, reward and over-commitment are negatively correlated with depersonalization (the correlation coefficients for effort, reward, and over-commitment with depersonalization are −0.28, −0.08, and −0.06, respectively, *p* < 0.01). Effort and over-commitment are positively with cynical, while reward is negatively correlated with cynical (the correlation coefficients for effort, reward, and over-commitment with professional efficacy are 0.54, −0.70, and 0.68, respectively, *p* < 0.01). Effort and over-commitment are positively correlated with burnout (the correlation coefficients for effort, over-commitment with burnout are 0.65 and 0.62, respectively, p < 0.01), while reward is negatively correlated with burnout (the correlation coefficients for reward with job burnout is −0.53, *p* < 0.01). ERI is positively correlated with burnout, emotional exhaustion, and cynical, while negatively correlated with depersonalization (the correlation coefficients for ERI with burnout and its three dimensions are 0.62, 0.62, 0.65, and −0.14, respectively, p < 0.01), with statistically significant differences. These results highlight a significant association between effort-reward imbalance and burnout, suggesting that interventions to reduce effort-reward imbalance could help alleviate burnout among emergency nurses. Additionally, the data suggest that higher over-commitment is closely associated with burnout. According to mediation effect testing principles, over-commitment may serve as a mediating factor in the relationship between occupational stress and burnout.

**Table 5:** Correlation analysis between ERI and burnout.

Variable	Emotional exhaustion	Cynical	Depersonalization	Job burnout	Effort	Over-commitment	Reward	ERI
Emotional exhaustion	1							
Cynical	0.878^**^	1						
Depersonalization	−0.096^**^	0.013	1					
Job burnout	0.876^**^	0.806^**^	−0.533^**^	1				
Effort	0.624^**^	0.535^**^	−0.280^**^	0.649^**^	1			
Over-commitment	0.684^**^	0.667^**^	−0.056^*^	0.619^**^	0.662^**^	1		
Reward	−0.629^**^	−0.695^**^	−0.081^**^	−0.534^**^	−0.501^**^	−0.759^**^	1	
ERI	0.624^**^	0.650^**^	−0.140^**^	0.623^**^	0.699^**^	0.712^**^	−0.799^**^	1

*P<0.05, **P<0.001.

### 3.6 Moderating effect of over-commitment

We firstly conducted univariate analysis of the variables, followed by multiple regression analysis to identify and include the appropriate control variables for the model. The results are presented in Supplementary materials. To further validate the moderating effect of over-commitment, a moderated mediation analysis was conducted using Model 1 from Hayes’ SPSS macro, as shown in the Table 6. The interaction term of ERI and over-commitment significantly predicted burnout (*β* = −0.31, *t* = −8.48, *p* < 0.001), with a Bootstrap 95% confidence interval of (−0.38, −0.24), indicating that over-commitment moderates the predictive effect of ERI on burnout (Table 6).

**Table 6:** Analysis of the moderating effects of the over-commitment.

Indicator	*β*	SE	*t*	95%*CI*	
				LLCI	ULCI
Constant	5.36	0.13	40.13^***^	5.10	5.62
ERI	7.12	0.47	15.29^***^	6.21	8.03
Over-commitment	0.30	0.04	7.83^***^	0.23	0.38
ERI × over-commitment	−0.31	0.04	−8.48^***^	−0.38	−0.24
*R*	0.69				
*R* ^2^	0.48				
*F*	463.72^***^				

^***^P < 0.001.

**Figure 1:**
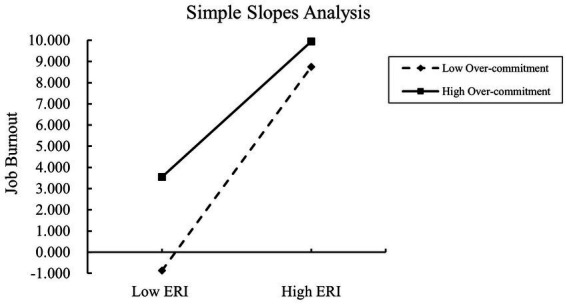
Simple slopes analysis.

Further, a simple slope analysis was conducted to examine the moderating effect of over-commitment at different levels (Figure 1). When over-commitment was low (M − 1SD), ERI had a significant positive moderating effect on burnout (simple slope = 8.38, *t* = 13.59, *p* < 0.001). However, for high over-commitment (M + 1SD), although ERI still had a positive moderating effect, the effect was weaker (simple slope = 5.57, *t* = 15.98, *p* < 0.001), suggesting that as over-commitment increases, the moderating effect of ERI on burnout decreases. Moreover, at three levels of over-commitment (M − 1SD, M, and M + 1SD), the moderating effect of ERI on burnout also showed a decreasing trend (Figure 1, Tables 7, 8). This suggests that as emergency nurses’ over-commitment decreases, ERI is more likely to contribute to burnout.

**Table 7:** The bootstrap method adjustment effect test of over-commitment at different levels.

Indicator	*β*	SE	*t*	*P*	95%*CI*	
					LLCI	ULCI
Constant	3.85	0.89	4.33	<0.001	2.11	5.59
ERI	6.98	0.47	14.84	<0.001	6.05	7.90
Over-commitment	0.30	0.04	7.61	<0.001	0.22	0.37
ERI × over-commitment	−0.30	0.04	−8.13	<0.001	−0.37	−0.23
Fertility status	−0.61	0.29	−2.13	0.03	−1.18	−0.05
Education	0.39	0.36	1.09	0.28	−0.31	1.09
Professional title	0.28	0.26	1.06	0.29	−0.24	0.80
Years of work	0.27	0.14	1.99	0.05	0.01	0.54
Weekly working hours	0.03	0.16	0.19	0.85	−0.28	0.34
Number of Night shifts	0.30	0.13	2.40	0.02	0.06	0.55
*R*	0.69					
*R* ^2^	0.48					
MSE	19.83					
*F*	156.88					
*P*	<0.001					

**Table 8:** The moderating effect of over-commitment at different levels.

Indicator	*β*	SE	*t*	*P*	95%CI	
					LLCI	ULCI
Eff1 (*M* − 1SD)	8.38	0.62	13.59	<0.001	7.17	9.59
Eff2 (*M*)	6.98	0.47	14.84	<0.001	6.05	7.90
Eff3 (*M* + 1SD)	5.57	0.35	15.98	<0.001	4.89	6.25

## 4 Discussion

This study explored the mediating effect of over-commitment in the relationship between external stress factors (effort-reward imbalance) and burnout in emergency nurses. The results showed that the ERI was 0.93 ± 0.57, and the overall burnout score was (4.77 ± 6.16). The ERI had a significant positive effect on burnout (*r* = 0.62, *p* < 0.001). Correlation analysis showed that the two dimensions of effort, over-commitment were positively correlated with burnout, with correlation coefficients of 0.65 and 0.62, respectively, reward and job burnout were negatively correlated, with correlation coefficients of −0.53, all significant. This indicates that effort, reward, and over-commitment independently predict burnout in emergency nurses. The significant positive correlation between ERI and burnout (*r* = 0.62) suggests that effort-reward imbalance can directly predict burnout and has a stronger predictive effect than either reward or over-commitment alone. The results emphasize the significant relationship between effort-reward imbalance and burnout, indicating that effort-reward imbalance in emergency nurses leads to more severe burnout problems, consistent with existing literature (23, 24). Healthcare workers are typically a group with high initial investment in their work, so the imbalance between effort and reward and the increase in over-commitment may exacerbate burnout in emergency nurses. Previous studies have shown that prolonged effort-reward imbalance can lead to lower job satisfaction, increased burnout (25), and higher risks of depression, cardiovascular diseases, and diabetes (26, 27). Nurses are often exposed to multiple professional challenges and dual physical and mental pressures arising from patient safety concerns and personal factors, which can easily lead them to a suboptimal health status (SHS). SHS is an intermediate condition between health and disease that cannot be diagnosed as a specific illness, characterized by persistent fatigue, weakness, reduced adaptability, and diminished physical function. Therefore, SHS not only affects nurses’ physical and mental health but may also compromise patient safety and further undermine the overall quality of hospital and healthcare services. Studies have shown that nurses with SHS have a significantly higher risk of experiencing effort-reward imbalance (ERI) and over-commitment compared to those in optimal health, high levels of effort-reward imbalance(ERI) and over-commitment make nurses more susceptible to suboptimal health status (28–31). Therefore, interventions aimed at reducing effort-reward imbalance may help alleviate burnout and reduce adverse health risks among emergency nurses.

Moderating effect analysis showed that when over-commitment was included in the model, the interaction term of ERI and over-commitment significantly predicted burnout (*β* = −0.31, *t* = −8.48, *p* < 0.001), indicating that external stress factors (effort and reward) not only directly affect burnout but also have an indirect effect through over-commitment. This suggests that over-commitment may mediate the health effects of effort and reward. Kinman and Jones (32) proposed that over-commitment, as an endogenous variable in the ERI model, can moderate the health-damaging or protective effects of effort and reward. The findings support the hypothesis that effort-reward imbalance influences burnout through the over-commitment dimension, consistent with research by Hinsch, Nuebling (33, 34), and others. Studies argued that high-risk occupational stress factors (e.g., work effort, responsibility, and workplace violence) can directly increase the risk of occupational health damage, while protective factors (e.g., income, respect, and recognition) moderate these effects (35, 36).

Simple slope analysis at different levels of over-commitment showed that as over-commitment increases, the moderating effect of ERI on burnout gradually decreases. Furthermore, at three levels of over-commitment (M − 1SD, M, and M + 1SD), the moderating effect of ERI on burnout decreased. This suggests that for emergency nurses with high over-commitment, burnout is exacerbated when their “over-commitment” is not adequately rewarded. In contrast, for emergency nurses with low over-commitment, their investment in work is relatively low, and their expectations of reward are also lower, meaning that effort-reward imbalance has a smaller impact on their burnout. For emergency nurses with high over-commitment, because they invest too much emotion and energy in their work, when their “over-commitment” does not receive appropriate returns, it will exacerbate their job burnout. While emergency nurses with low over-commitment have relatively less investment in their work, and their expectations for returns are also relatively low, so the impact of imbalance between input and return on their job burnout is relatively small. Emergency nurses consume physical and mental resources during the work process, and without rest for a long time, there will be a negative cumulative effect, requiring a certain amount of recovery time. However, individuals with a higher level of over-commitment often find it difficult to effectively detach from work (37, 38), thus running the risk of depleting their physical and mental resources. High levels of ERI and over-commitment are highly likely to lead to job burnout, which serves as a significant predictor of psychological symptoms such as depression and post-traumatic stress disorder. Social withdrawal is correlated with depression, and prolonged social withdrawal can exacerbate mental health issues by weakening stress coping mechanisms, reducing life satisfaction, and contributing to cognitive decline. Nurses face a high risk of mental health problems such as depression, anxiety, and stress, and their psychological well-being directly impacts the quality of healthcare and nursing services (39–47).

### 4.1 Limitation

This study is a cross-sectional survey, which cannot predict the causal relationships between variables. Secondly, reliance on self-reported data may introduce potential biases. While we fully acknowledge the originality of the data, statistical analyses could still be subject to bias. Furthermore, this study focused on emergency department nurses to investigate the moderating effect of over-commitment in the relationship between ERI and burnout, which may not fully capture variations across different clinical departments. It is recommended that future research could consider using a longitudinal design to clarify the relationship between effort-reward imbalance and nurse burnout, and verify these results in nursing populations across various clinical departments.

## 5 Conclusion

In conclusion, this study explored the moderating effect of over-commitment in the relationship between effort-reward imbalance and burnout among emergency nurses. The results show that effort-reward imbalance can have a moderating effect on burnout through over-commitment, and that as over-commitment increases, the moderating effect of effort-reward imbalance on burnout decreases. This study contributes to a deeper understanding of the relationship between effort-reward imbalance and burnout in emergency nurses and provides a theoretical foundation for future research. In practice, management should focus more on the issue of effort-reward imbalance among emergency nurses and provide support to those experiencing this imbalance to alleviate burnout caused by occupational stress. Such as conducting regular psychological assessments and providing targeted psychological interventions, optimizing scheduling systems, recognizing employees’ efforts and offering appropriate rewards, and providing more opportunities for further education and career advancement. Additionally, it is important to recognize the phenomenon of excessive work involvement among healthcare workers, encouraging them to work diligently while also preventing excessive over-commitment from leading to burnout and mental health issues. Nursing managers should propose differentiated interventions for nurses with low over-commitment, focus on monitoring workload-reward balance and enhancing organizational support; for those with high over-commitment, guide them in setting work boundaries, implementing flexible task allocation, and providing stress-management training to prevent implicit exhauon, and providing stress-management training to prevent implicit exhaustion. Nursing managers should consider system-level psychological assessments and dynamic monitoring, which could also enable early identification and targeted resource allocation, thereby improving the nursing team’s resilience and sustainable functioning.

## Data Availability

The original contributions presented in the study are included in the article/Supplementary materials, further inquiries can be directed to the corresponding author/s.
