# Supplementary material for: The association between effort-reward imbalance and job burnout among emergency nurses: the moderating effect of over-commitment
Source: Front Public Health. 2026 Jan 16;13:1707511. doi: 10.3389/fpubh.2025.1707511 (PMC12856932; doi:10.3389/fpubh.2025.1707511)
Supplement: Supplementary file 2 [file Data_Sheet_2.pdf]

Table Univariate analysis of Variable

Variable	Category	*t/F*	*P*
Sex	Female	0.92	0.36
	Male		
Marital status	Single	-2.89	0.01
	Married		
Fertility Status	No	-2.09	0.04
	Yes		
Education	Junior college degree	-4.47	< 0.001
	Bachelor's degree or above		
Professional title	Junior	15.61	< 0.001
	Intermediate		
	Senior		
Years of work（year）	≤5	6.13	< 0.001
	6-10		
	11-15		
	16-20		
	> 20		
Weekly working hours（h/week）	< 40	13.46	< 0.001
	41-48		
	49-58		
	≥59		
Number of Night shifts per month	0 times/week	7.24	< 0.001
	1-4 times/week		
	5-8 times/week		
	≥9 times/week		
Monthly income	< 4000 yuan/month	1.06	0.38
	4000-5999yuan/month		
	6000-7999yuan/month		
	8000-9999yuan/month		
	≥10000yuan/month		
Smoking situation	Do not smoke	-0.80	0.42
	Smoking		
Alcohol consumption situation	Do not drink alcohol	-1.12	0.26
	Drinking		

Table Multivariate linear regression analysis of Variable

variable	*B*	*SE*	*P*	95%CI		Tolerance	VIF
				LLCI	ULCI		
Marital status	0.70	0.53	0.19	-0.34	1.74	0.35	2.83
Fertility Status	-1.19	0.54	0.03	-2.24	-0.14	0.32	3.10
Education	1.71	0.48	< 0.001	0.77	2.64	0.96	1.05
Professional title	1.08	0.35	0.01	0.39	1.77	0.58	1.74
Years of work	0.57	0.18	0.01	0.21	0.93	0.44	2.26
Weekly working hours	1.17	0.20	< 0.001	0.77	1.57	0.98	1.02
Number of Night shifts	0.74	0.17	< 0.001	0.41	1.07	0.88	1.13
R	0.26a						
R^2	0.07						
Adjusted R^2	0.07						
Change in R^2	0.07						
F	16.26						
P	0.001						

a Dependent variable: Burnout; a Predictor variable: Marital status, Fertility Status, Education, Professional title, Years of work, Weekly working hours, Number of Night shifts
