# Supplementary material for: The association between effort-reward imbalance and job burnout among emergency nurses: the moderating effect of over-commitment
Source: Front Public Health. 2026 Jan 16;13:1707511. doi: 10.3389/fpubh.2025.1707511 (PMC12856932; doi:10.3389/fpubh.2025.1707511)
Supplement: Supplementary file 3 [file Data_Sheet_3.pdf]

Normality test

	Kolmogorov-Smirnov a test			Shapiro-Wilk test		
	Statistics	Degrees of freedom	Significance	Statistics	Degrees of freedom	Significance
Emotional exhaustion	0.11	1540	0	0.946	1540	0
Cynical	0.136	1540	0	0.897	1540	0
Depersonalization	0.099	1540	0	0.948	1540	0
Job burnout	0.087	1540	0	0.97	1540	0
Effort	0.088	1540	0	0.975	1540	0
Over-commitment	0.105	1540	0	0.959	1540	0
Reward	0.089	1540	0	0.944	1540	0
ERI	0.192	1540	0	0.694	1540	0

a Lilliefors test
